# Automatic segmentation of hepatic metastases on DWI images based on a deep learning method: assessment of tumor treatment response according to the RECIST 1.1 criteria

**DOI:** 10.1186/s12885-022-10366-0

**Published:** 2022-12-07

**Authors:** Xiang Liu, Rui Wang, Zemin Zhu, Kexin Wang, Yue Gao, Jialun Li, Yaofeng Zhang, Xiangpeng Wang, Xiaodong Zhang, Xiaoying Wang

**Affiliations:** 1 Department of Radiology, Peking University First Hospital, No.8 Xishiku Street, Xicheng District, Beijing, 100034 China; 2 Department of Hepatobiliary and Pancreatic Surgery, Zhuzhou Central Hospital, Zhuzhou, 412000 China; 3 School of Basic Medical Sciences, Capital Medical University, Beijing, 100069 China; 4 Beijing Smart Tree Medical Technology Co. Ltd., Beijing, 100011 China

**Keywords:** Deep learning, RECIST 1.1 criteria, Liver metastases, DWI

## Abstract

**Background:**

Evaluation of treated tumors according to Response Evaluation Criteria in Solid Tumors (RECIST) criteria is an important but time-consuming task in medical imaging. Deep learning methods are expected to automate the evaluation process and improve the efficiency of imaging interpretation.

**Objective:**

To develop an automated algorithm for segmentation of liver metastases based on a deep learning method and assess its efficacy for treatment response assessment according to the RECIST 1.1 criteria.

**Methods:**

One hundred and sixteen treated patients with clinically confirmed liver metastases were enrolled. All patients had baseline and post-treatment MR images. They were divided into an initial (*n* = 86) and validation cohort (*n* = 30) according to the examined time. The metastatic foci on DWI images were annotated by two researchers in consensus. Then the treatment responses were assessed by the two researchers according to RECIST 1.1 criteria. A 3D U-Net algorithm was trained for automated liver metastases segmentation using the initial cohort. Based on the segmentation of liver metastases, the treatment response was assessed automatically with a rule-based program according to the RECIST 1.1 criteria. The segmentation performance was evaluated using the Dice similarity coefficient (DSC), volumetric similarity (VS), and Hausdorff distance (HD). The area under the curve (AUC) and Kappa statistics were used to assess the accuracy and consistency of the treatment response assessment by the deep learning model and compared with two radiologists [attending radiologist (R1) and fellow radiologist (R2)] in the validation cohort.

**Results:**

In the validation cohort, the mean DSC, VS, and HD were 0.85 ± 0.08, 0.89 ± 0.09, and 25.53 ± 12.11 mm for the liver metastases segmentation. The accuracies of R1, R2 and automated segmentation-based assessment were 0.77, 0.65, and 0.74, respectively, and the AUC values were 0.81, 0.73, and 0.83, respectively. The consistency of treatment response assessment based on automated segmentation and manual annotation was moderate [*K* value: 0.60 (0.34–0.84)].

**Conclusion:**

The deep learning-based liver metastases segmentation was capable of evaluating treatment response according to RECIST 1.1 criteria, with comparable results to the junior radiologist and superior to that of the fellow radiologist.

## Background

About 5% of newly diagnosed cancer patients presented with synchronous liver metastases and the presence of liver metastasis was associated with reduced survival [1]. Metastases in the liver are typically treated with systemic chemotherapy, ablation, and surgery, depending on the source and stage [2].

Radiological assessment of the treatment response is often a prerequisite to clinical decisions in cancer treatment [3]. Image-based evaluation, using either computed tomography (CT) or magnetic resonance imaging (MRI) images, can noninvasively visualize the tumor during the treatment. Compared with CT, liver magnetic resonance imaging (MRI) is superior for hepatic metastasis evaluation [4, 5]. Diffusion-weighted imaging (DWI)-related parameters are appealing as imaging biomarkers, and DWI alone might be used for tumor evaluation with excellent performance [6].

Response Evaluation Criteria in Solid Tumor 1.1 (RECIST 1.1) is accepted as a standard method and widely used clinical guideline for the evaluation of response and progress of solid tumors [7, 8]. The application of the RECIST1.1 guideline involves a series of tumor size measurements, which is an important surrogate marker of therapeutic efficacy [9]. Consistent and accurate measurements of the tumor size are essential with their direct impact on cancer treatment management.

However, performing RECIST measurement is a non-trivial task requiring a great deal of expertise and time by a highly trained radiologist. Multiple reports have indicated that the tumor size measurements are subject to intra- and interobserver variability, with various environmental factors causing the variability [10, 11]. To address these challenges, researchers have attempted to develop computer-aided systems to assist in lesion measurement through automated lesion segmentation [12, 13].

Therefore, in this study, we proposed a deep learning-based liver metastases segmentation method to assess the treatment response on DWI images according to the RECIST1.1 criteria. The objective of this study was to assess the feasibility and accuracy of the automated treatment response assessment by comparison between different reading levels of radiologists.

## Materials and methods

### Study design

This study was approved by the local institutional review board and informed consent was waived according to its retrospective design. The study population included the initial cohort and validation cohort. The initial cohort (2017.1–2020.12) was used to develop the deep learning-based liver and liver metastases segmentation algorithms. The validation cohort (2021.1–2022.3) was used to validate the performance of the segmentation models and their accuracy in treatment response assessment of hepatic metastasis.

### Patient enrollment

Two hundred and three patients with histologically confirmed primary cancer (colorectal cancer, gastrointestinal cancer, pancreatic cancer, and so on) who underwent curative treatment of liver metastases were included in this study between Jan 2017 and Mar 2022. All patients underwent abdominal MRI before the start (baseline) and after the end of at least one-circle treatment (post-treatment).

**Fig. 1:**
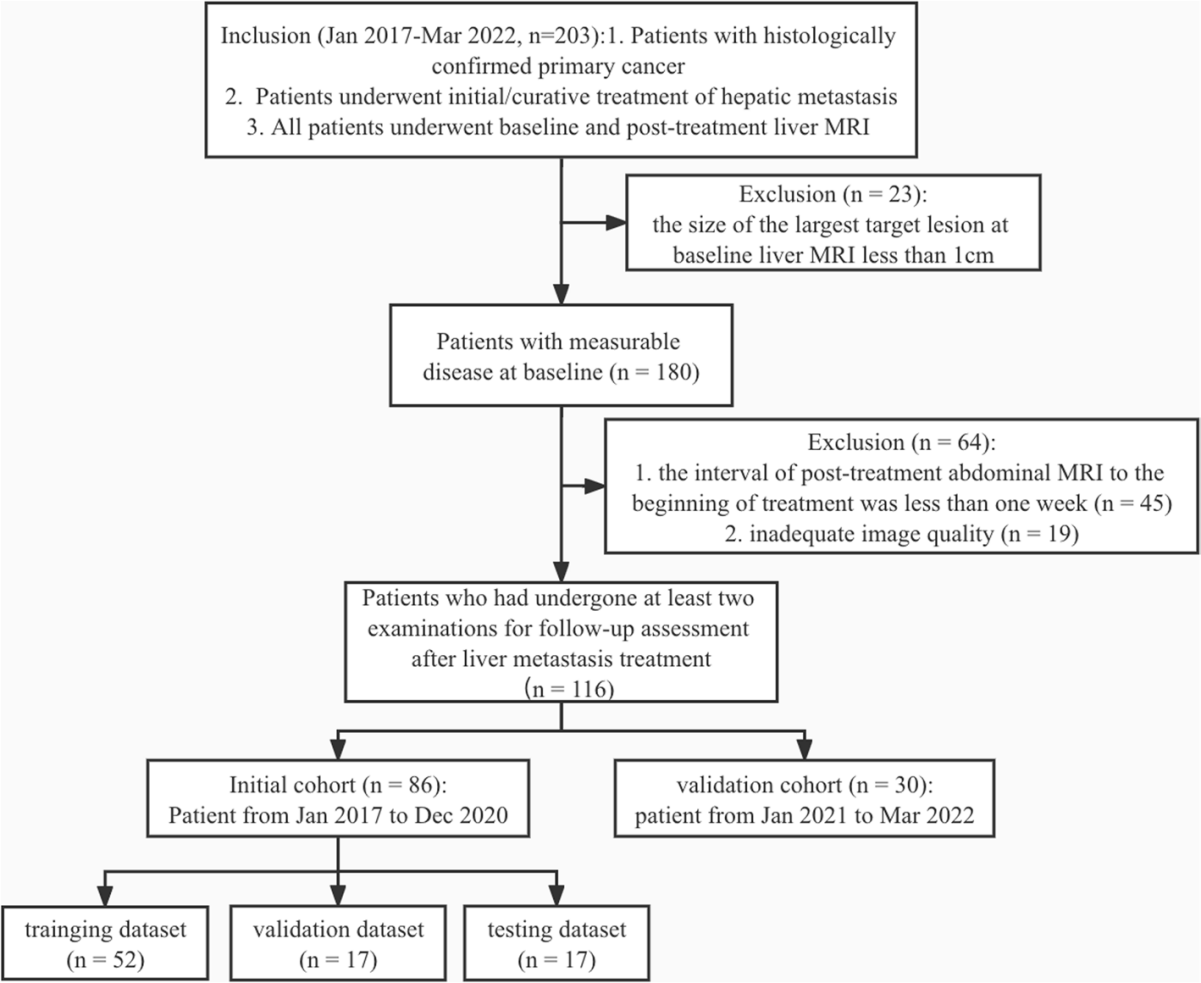
The flowchart of patient enrollment

According to the RECIST1.1 criteria, only patients with measurable disease at baseline MRI should be included in protocols. Hence, 23 of the 203 patients were excluded because of no measurable liver metastasis (the largest diameter of the lesions < 1 cm). In addition, 45 patients were excluded due to the interval of post-treatment abdominal MRI to the beginning of treatment being less than one week; and nine patients were excluded for the inadequate image quality. Finally, 116 patients who had undergone at least two scans for follow-up assessment after liver metastases treatment were analyzed (Fig. 1). Demographic and clinical features of the enrolled patients were acquired from the electronic information system, including gender, age, number of metastatic lesions, location of primary cancer, and treatment methods.

### MRI acquisition

Abdominal MRI scans were performed using one of the three 3.0 T magnet scanners (Achieva, Philips Healthcare; Discovery MR750, GE Healthcare; Intera, Philips Healthcare) with body phased-array coils. The following sequences were performed as the liver MRI protocol: (1) axial respiratory-triggered T2-weighted imaging (T2WI) with fat suppression turbo spin-echo sequence; (2) axial in- and opposed-phase T1-weighted imaging (T1WI) of gradient echo sequence; (3) axial DWI of single-shot echo-planar sequence with automatically generated apparent diffusion coefficient (ADC) maps; and (4) axial multiphase dynamic contrast-enhanced (DCE) T1WI sequence. Detailed scanning parameters of T2WI, DWI and DCE are listed in Table 1.

**Table 1:** Parameters of the main MRI sequences

Parameters	Achieva, Philips			Discovery GE			Intera, Philips		
Sequences	T2WI	DWI	DCE	T2WI	DWI	DCE	T2WI	DWI	DCE
Repetition time (ms)	2640	4250	6.7	2520	4000	3.9	2765	4959	7.5
Echo time (ms)	100	76	2.4	95	60	2.0	87	78	2.4
Flip angle (degree)	-	-	10	-	-	13	-	-	13
Field of view (mm)	280 × 220	280 × 220	400 × 400	250 × 200	250 × 200	450 × 360	250 × 200	250 × 200	450 × 350
Matrix size	156 × 180	156 × 180	280 × 180	256 × 256	256 × 256	288 × 192	240 × 240	240 × 240	320 × 200
Section thickness (mm)	4	4	3	5	5	3	4	4	2
Intersection gap (mm)	1	1	0	1	1	0	1	1	0
*b*-values (s/mm^2^)	-	1000	-	-	1400	-	-	1400	-

*DCE* Dynamic contrast-enhanced, *DWI* Diffusion weighted imaging, *T1WI* T1-weighted imaging, *T2WI* T1-weighted imaging

### Manual annotation of liver and liver metastases

The annotation of the liver and the liver metastases foci were performed using an open-source software platform (ITK-SNAP, version3.6.0-RC1; http://www.itksnap.org). Under the supervision of a board-certified radiology expert (with more than 20 years of reading experience), a radiology resident with three years of reading experience evaluated all MRI examinations and, section by section manually annotated the liver and liver metastases on DWI images. Areas containing air, obvious vascular structures, and artifacts were avoided.

The reference standard for liver metastases was a histological result, or the metastatic lesions were proved by clinical comprehensive information (employing imaging, serum tumor markers, and the follow-up outcome). The typical imaging appearances of liver metastases involved: hyperintense on high b-value DWI images; moderately hyperintense to the surrounding liver parenchyma on T2WI images; hyper-vascular or peripherally enhanced on DCE T1WI images. The metastatic lesions were annotated on the DWI images, covering all tumor areas, including areas of necrosis and fibrosis. The target lesions were recognized by the two radiologists and were measured to assess the treatment responses according to RECIST 1.1 criteria.

### Model development of liver and liver metastases segmentation

**Fig. 2:**
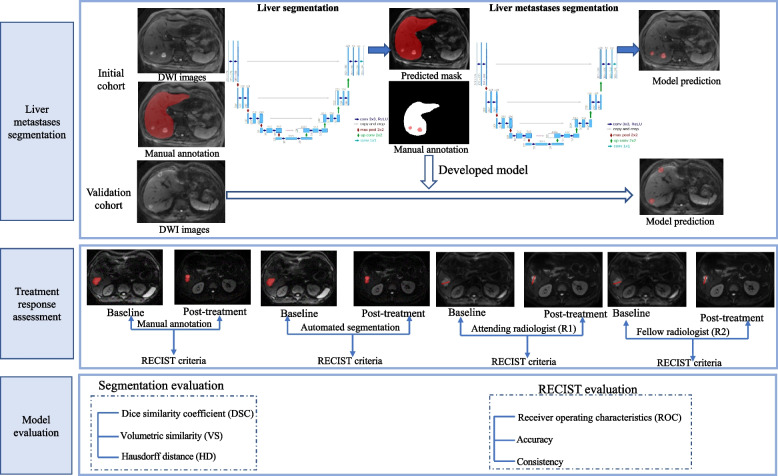
The flowchart of model development and evaluation

The segmentation framework consisted of two components: liver segmentation and metastases segmentation from the liver region. A deep learning-based 3D U-Net was first developed to automatically perform liver segmentation in both the baseline and the follow-up MRI scans, then followed by a second step with a 3D U-Net for liver metastases segmentation within the segmented liver mask (Fig. 2).

Regarding the model development of liver segmentation, 86 patients with were randomly divided into either the training (*n* = 52), validation (*n* = 17), or testing (*n* = 17) datasets with a ratio of 6:2:2 in the initial cohort. All the input images of DWI were unified and resized to 224 × 224 × 64 (z, y, x) before training to maintain the optimal image features, and z-score intensity normalization was applied to all images. Skewing (angel: 0–5), shearing (angel: 0–5) and translation (scale: -0.1,0.1) of the images were applied for data augmentation. The training was carried out over 300 epochs using an Adam Optimizer with a learning rate of 0.01, a batch size of 2, and a dice loss function. During model development, other hyperparameters (such as weight initialization and dropout for regularization) were randomly selected and automatically executed.

The volume of interest in the liver predicted by the liver segmentation model was used as the mask for the liver metastases segmentation. The model development parameters and network configurations for metastases segmentation were the same as the liver segmentation model. Both the CNNs were coded by Python3.6, Pytorch 0.4.1, Opencv, Numpy, and SimpleITK, and trained on the GPU NVIDIA Tesla P100 16G.

### Treatment response assessment

The outcomes of the treatment response assessment came from four sources, i.e., the reference standard, the automatic, and the two radiologists. They assessed the images according to RECIST 1.1 criteria [14], including complete response (CR), partial response (PR), stable disease (SD), and progressive disease (PD).

The reference standard of treatment response assessment was given by the radiologists who made the manual annotations. The automatic assessment was given by the model. It was based on the automated segmentation of liver metastases on DWI images, the diameters of the lesions were calculated and the assessments were then given by a rule-based program. On the baseline DWI images, the lesions with the longest diameter of more than 10 mm were regarded as measurable. Up to 5 the largest of the measurable lesions were chosen as the target lesions. On the post-treatment DWI images, the number of the target lesions was calculated and compared with that of the baseline images. The increase in the number of target lesions indicated the appearance of new lesions (classified as PD). The sum of the longest diameters of a maximum of five target lesions in each patient was computed on baseline and post-treatment DWI images, and the percentage change of the total length between lesions on post-treatment and baseline DWI was computed for treatment response assessment.

In addition, two radiologists with different levels of experience (an attending radiologist [R1] and a fellow radiologist [R2] with 8- and 4 years’ experience in abdominal imaging, respectively) independently measured the maximum diameter of the target metastases and evaluated the treatment response with access to the full examinations according to the RECIST 1.1criteria.

### Statistical analysis

The “mean ± standard deviation (SD)” values are used for the description of continuous variables with normal distribution. Descriptive statistics of the categorical data are presented with “n (%)”. The independent t-test and Chi-square test were applied to determine the difference of continuous (age, lesion size, lesion volume, and ADC values) and categorical variables (gender, location of the primary tumor, etc.), respectively, in the initial cohort and validation cohort. In the testing dataset and validation cohort, the evaluation metrics used for the liver and liver metastases segmentation included the overlap-based metric [Dice similarity coefficient (DSC)], the volume-based metric [volumetric similarity (VS)], and the spatial distance-based metric [Hausdorff distance (HD)] [15].

Receiver operating characteristics (ROC) curve and area under the curve (AUC) were used to assess the accuracy of treatment response assessment. The kappa statistics were applied for the consistent evaluation of treatment response in both initial and validation cohorts. A *P*-value less than 0.05 was treated as significant. Statistical analysis was performed with MedCalc (version 14.8; MedCalc Software, Ostend, Belgium) and R version 3.4.1.

## Results

### Study population

In this study, 116 eligible patients with liver metastases were included. These patients were divided into two cohorts according to scanning time: 86 patients (48/86 male, 38/86 female, mean age 60 years, range 32–82 years) constituted an initial cohort; and 30 patients (19/30 male, 11/30 female, mean age 60 years, range 35–72 years) constituted the validation cohort. 37% of the patients (32/86) and 20% of the patients (6/30) exhibited more than five liver target lesions in the two cohorts, respectively. The baseline characteristics of the enrolled patients are shown in Table 2. It showed no significant differences between the initial and validation cohorts regarding demographic and clinical characteristics.

**Table 2:** Main baseline demographics and clinical characteristics of patients in the cohorts

	Initial cohort					Validation cohort	*P* value
Characteristics	Training dataset (*n* = 52)	Validation dataset (*n* = 17)	Testing dataset (*n* = 17)	*P* value	Total (*n* = 86)	(*n* = 30)	
Age(y)	61 ± 10	58 ± 11	59 ± 11	0.737	60 ± 11	59 ± 10	0.592
Gender [N (%)]				0.398			0.071
Male	21(40.38%)	10 (58.82%)	7 (41.18%)		38 (44.19%)	19 (63.33%)	
Female	31(59.62%)	7 (41.18%)	10 (58.82%)		48 (55.81%)	11 (36.67%)	
Location of the primary tumor [N (%)]				0.992			0.135
Rectal cancer	10 (19.23%)	4 (23.53%)	2 (11.76%)		16 (18.60%)	8 (26.67%)	
Colon cancer	16 (30.77%)	5 (29.41%)	6 (35.29%)		27 (31.40%)	7 (23.33%)	
Breast cancer	8 (15.38%)	2 (11.76%)	2 (11.76%)		12 (13.95%)	3 (10.00%)	
Renal cancer	4 (7.70%)	1 (5.88%)	1 (5.88%)		6 (6.98%)	5 (16.67%)	
Prostate cancer	3 (5.77%)	0 (0.00%)	2 (11.76%)		5 (5.81%)	0 (0.00%)	
Small bowel cancer	3 (5.77%)	1 (5.88%)	1 (5.88%)		5 (5.81%)	3 (10.00%)	
Lung cancer	2 (3.85%)	2 (11.76%)	1 (5.88%)		5 (5.81%)	4 (13.33%)	
Others	6 (11.54%)	2 (11.76%)	2 (11.76%)		10 (11.63%)	0 (0.00%)	
Number of target lesions [N (%)]				0.345			0.006
1	14 (26.92%)	2 (11.76%)	6 (35.29%)		22 (25.58%)	8 (9.30%)	
2	7 (13.46%)	4 (23.53%)	1 (5.88%)		12 (13.95%)	4 (4.65%)	
3	4 (7.69%)	2 (11.76%)	0 (0.00%)		6 (6.98%)	10 (11.63%)	
4	6 (11.54%)	3 (17.65%)	5 (29.41%)		14 (16.28%)	2 (2.33%)	
≥ 5	21 (40.38%)	6 (35.29%)	5 (29.41%)		32 (37.21%)	6 (6.98%)	
Baseline lesion size (cm)	6.6	7.0	5.6	0.813	6.5	4.7	0.126
Baseline lesion volume (cm^3^)	308.51	214.70	55.83	0.801	240.02	61.37	0.615
ADC value of baseline lesion (mm^2^/s)	1.1	1.2	1.2	0.903	1.1	1.0	0.090

*ADC* Apparent diffusion coefficient

### Treatment protocol

The treatment protocols of all patients for liver metastases were followed systematically. Fifty-four patients (46.55%) received chemotherapy only, 15 patients (12.93%) received surgery/ radiofrequency ablation only, and 47 patients (36.21%) received a combination of surgery/RFA and chemotherapy. Five chemotherapy protocols were included in this study: Cetuximab + FOLFOX (*n* = 36; 35.64%); Bevacizumab + XELIRI (*n* = 29; 28.71%); Etoposide + carboplatin + natalizumab (*n* = 20; 19.80%); Bevacizumab + Xeloda (*n* = 10; 9.90%); Gemcitabine + albumin + paclitaxel (*n* = 6; 5.94%).

In addition, all patients had received at least one course of post-treatment MRI examination for liver metastases in both initial and validation cohorts. In the initial cohort, 55 patients had one post-treatment examination, 15 patients had two post-treatment examinations, 9 patients had three post-treatment examinations, 3 patients had four post-treatment examinations, 1 patient had five post-treatment examinations, 2 patients had seven post-treatment examinations and 1 patient had nine post-treatment examinations; in the validation cohort, 29 patients had one post-treatment examination, 1 patient had two post-treatment examinations. The detailed examination protocol was shown in Table 3.

**Table 3:** The MRI examination protocols

	Initial cohort			Validation cohort		
Time of MRI	No. patients	No. lesions	Total of MRI scans	No. patients	No. lesions	Total of MRI scans
Baseline	86	676	86	30	132	30
1^st^ post-treatment	86	651	172	30	138	60
2^nd^ post-treatment	31	245	203	1	9	61
3^rd^ post-treatment	16	106	219	-	-	-
4^th^ post-treatment	7	56	226	-	-	-
5^th^ post-treatment	4	24	230	-	-	-
6^th^ post-treatment	3	16	233	-	-	-
7^th^ post-treatment	3	14	236	-	-	-
8^th^ post-treatment	1	7	237	-	-	-
9^th^ post-treatment	1	7	238	-	-	-

### Assessment of liver and liver metastasis segmentation

**Fig. 3:**
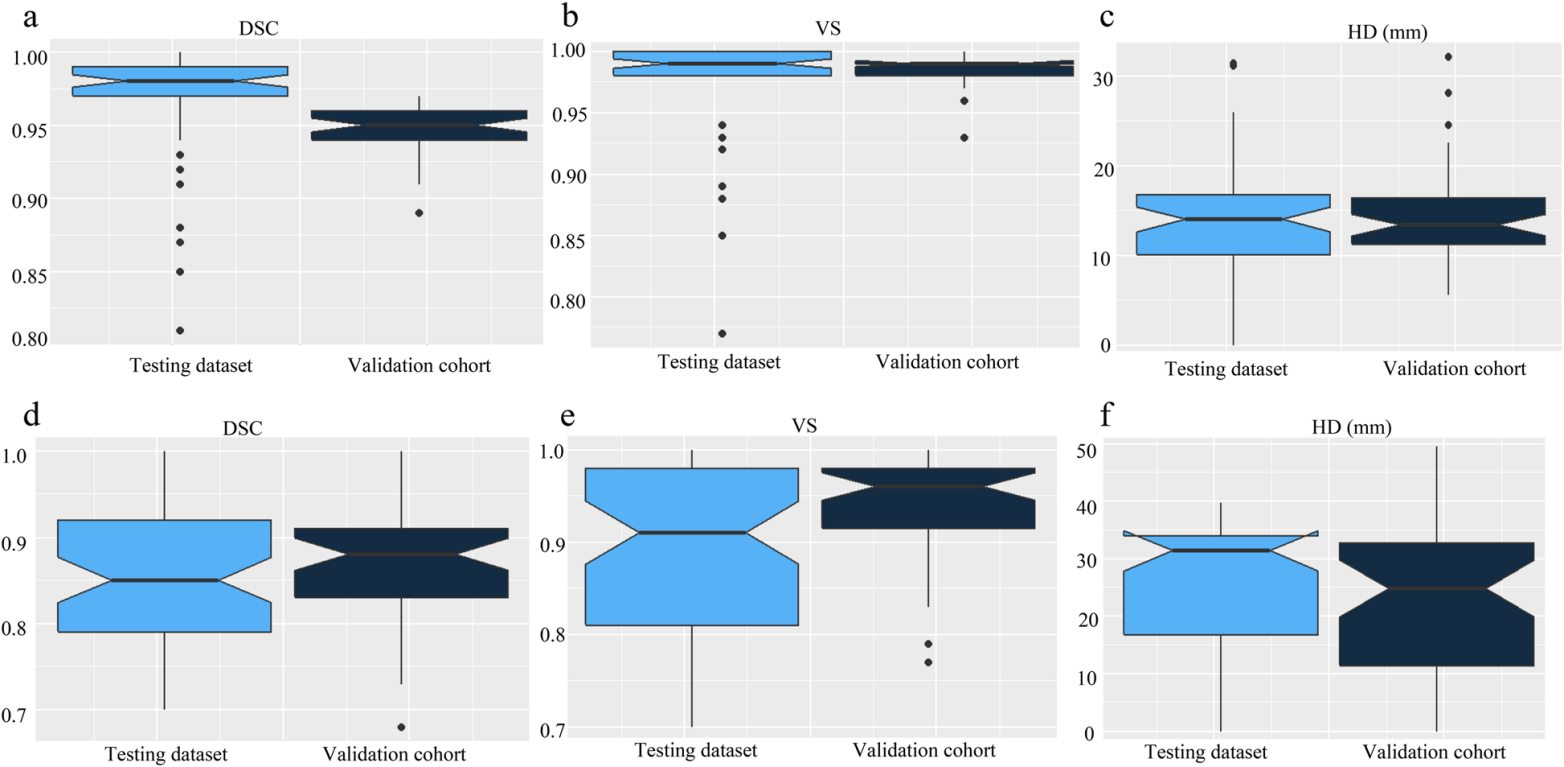
Notched box plots of the segmentation results in the testing dataset and validation cohort. **a-c** the DSC, VS, and HD of liver segmentation; d-f: the DSC, VS and HD of liver metastases segmentation. DSC: Dice similarity coefficient; HD: Hausdorff distance; VS: Volumetric similarity

Seventeen patients with 47 abdominal MRI scans total and 30 patients with 61 abdominal MRI scans were analyzed in the testing dataset and validation cohort, respectively. As shown in Fig. 3 and Table 4, the mean DSC, VS and HD for the automatic liver segmentation are 0.95 ± 0.16, 0.98 ± 0.01, 14.39 ± 5.15 mm in the testing dataset and 0.97 ± 0.04, 0.97 ± 0.04, 13.39 ± 7.47 mm in the validation cohort (Fig. 3a-c). The mean DSC, VS and HD for the automatic liver metastases segmentation are 0.87 ± 0.07, 0.94 ± 0.06, 22.67 ± 13.83 mm in the testing dataset and 0.85 ± 0.08, 0.89 ± 0.09, 25.53 ± 12.11 mm in the validation cohort (Fig. 3d-f). In a subgroup analysis, the segmentation results between patients with more than five target lesions or not were compared, which showed no significant difference in both the testing dataset and validation cohort.

**Table 4:** The segmentation results of liver and liver metastases in the testing dataset and validation cohort

	Testing dataset				Validation cohort			
	Target Lesions < 5	Target Lesions ≥ 5	*P value*	all	Target Lesions < 5	Target Lesions ≥ 5	*P value*	all
Liver segmentation								
DSC	0.95 ± 0.02	0.95 ± 0.02	0.976	0.95 ± 0.16	0.97 ± 0.04	0.96 ± 0.04	0.116	0.97 ± 0.04
VS	0.98 ± 0.01	0.98 ± 0.01	0.465	0.98 ± 0.01	0.98 ± 0.04	0.97 ± 0.04	0.122	0.97 ± 0.04
HD (mm)	15.34 ± 5.54	13.15 ± 4.43	0.635	14.39 ± 5.15	12.98 ± 7.51	14.30 ± 7.52	0.617	13.39 ± 7.47
Liver metastases segmentation								
DSC	0.88 ± 0.07	0.88 ± 0.08	0.433	0.87 ± 0.07	0.86 ± 0.09	0.84 ± 0.09	0.836	0.85 ± 0.08
VS	0.94 ± 0.05	0.94 ± 0.06	0.477	0.94 ± 0.06	0.90 ± 0.10	0.88 ± 0.09	0.814	0.89 ± 0.09
HD (mm)	20.11 ± 14.93	25.99 ± 11.78	0.065	22.67 ± 13.83	23.81 ± 12.86	26.31 ± 9.48	0.081	25.53 ± 12.11

*DSC* Dice similarity coefficient, *VS* Volumetric similarity, *HD* Hausdorff distance

### Accuracy of the treatment response assessment

**Fig. 4:**
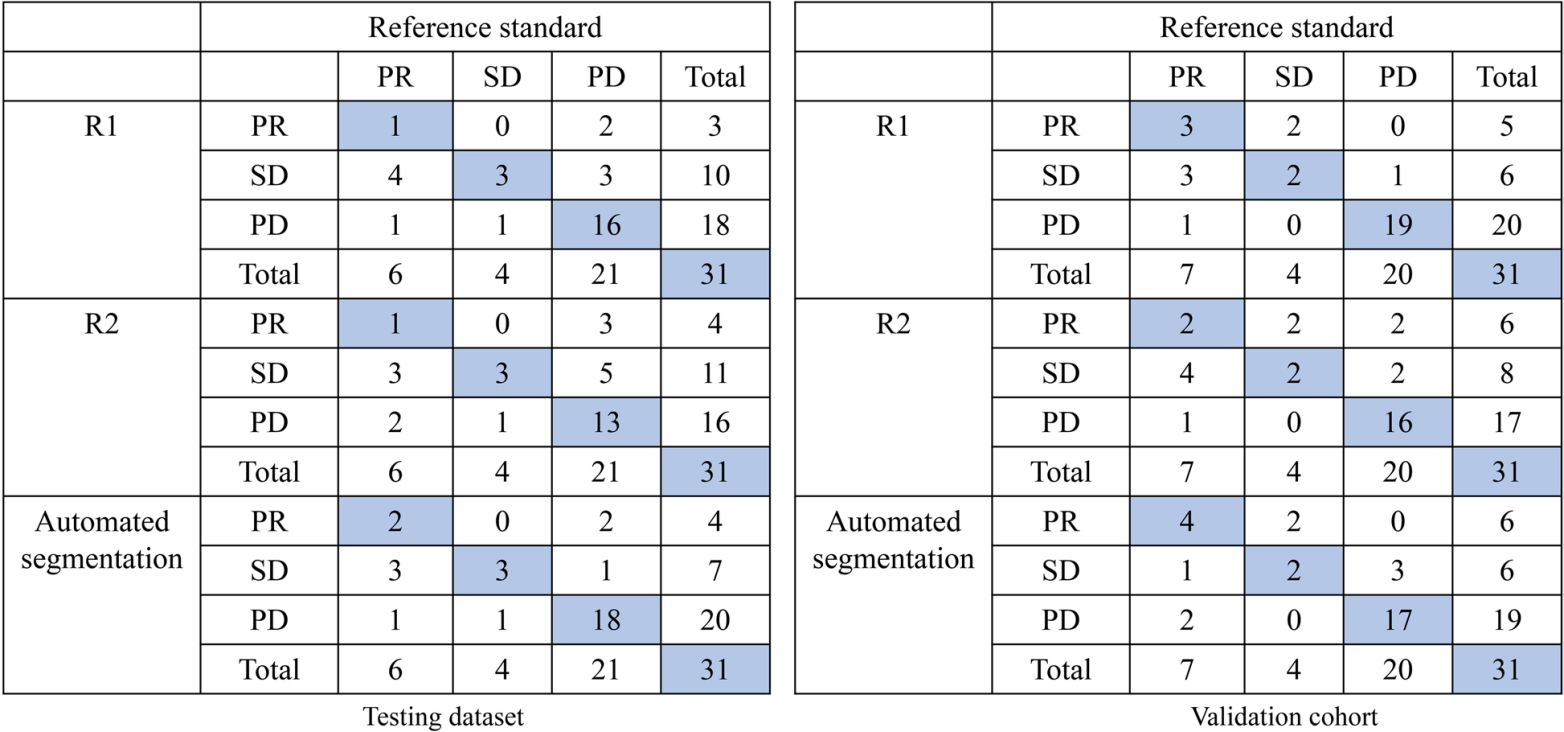
The confusion matrix of the response assessment results with respect to reference standard. R1: attending radiologist; R2: fellow radiologist

**Fig. 5:**
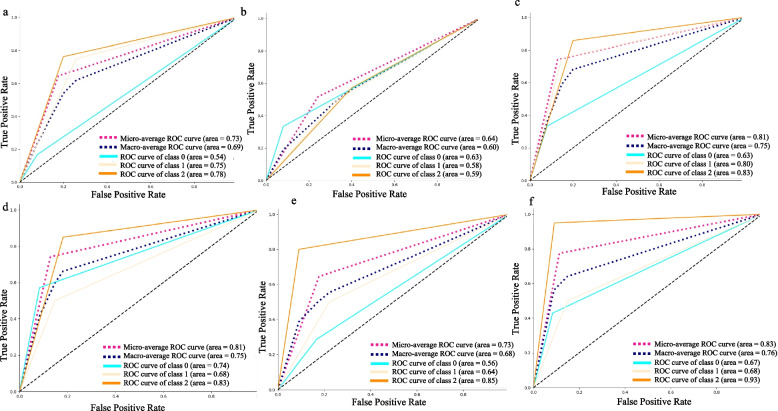
Receiver operating characteristic curves (ROC) for the therapy response assessment. **a** attending radiologist (R1) in the testing dataset; **b** fellow radiologist (R2) in the testing dataset; **c** automated segmentation-based assessment in the testing dataset; **d** R1 in the validation cohort; **e** R2 in the validation cohort; **f** automated segmentation-based assessment in the validation cohort

**Fig. 6:**
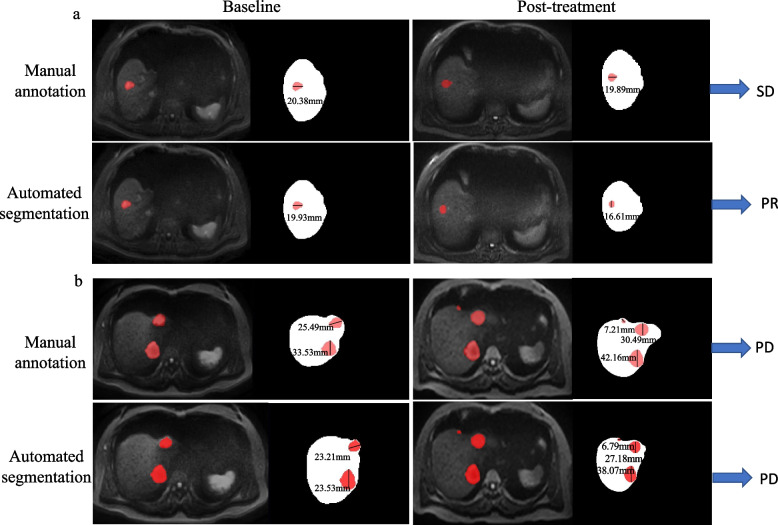
Example results of the treatment response assessment on DWI image. **a** liver metastasis from breast cancer in a 55-year -old female patient who was classified as having stable disease based on the manual liver metastasis segmentation but having partial response based on the automated liver metastasis segmentation; **b** liver metastases from rectal cancer in a 67-year-old male patient who was classified as showing progressive disease on the basis of manual and automated liver metastases segmentation

Seventeen patients with 31 pairs of abdominal MRI scans and 30 patients with 31 pairs of abdominal MRI scans were analyzed in the testing dataset and validation cohort, respectively. The response assessment results in the testing dataset and validation cohort are shown in Fig. 4 using a confusion matrix. According to the confusion matrix, the accuracies of R1, R2 and automated segmentation-based response assessment were 0.64 (95%CI: 0.47–0.79), 0.54 (95%CI: 0.38–0.71), and 0.74 (95%CI: 0.57–0.87) in the testing cohort (*P* values: R1*vs.* R2: 0.001; R1 *vs.* automated segmentation: 0.001; R2 *vs.* automated segmentation: 0.025) and 0.77 (95%CI: 0.60–0.89), 0.65 (95%CI: 0.47–0.79), and 0.74 (95%CI: 0.57–0.87) in the validation cohort (*P* values: R1*vs.* R2: 0.001; R1 *vs.* automated segmentation: 0.051; R2 *vs.* automated segmentation: 0.001). Figure 5 showed the ROC plots in the testing dataset and validation cohort, and the AUC values of R1, R2, and automated segmentation-based assessment were 0.73, 0.64, and 0.83, respectively, in the testing dataset, and 0.81, 0.73, 0.83, respectively, in the validation cohort. Example results of the treatment response assessment based on manual and automated segmentation are shown in Fig. 6.

### Consistency of the treatment response assessment

As shown in Table 5, the agreement of treatment response assessment based on automated segmentation and reference standard was moderate [*K* value: 0.51 (0.23–0.79)] in the testing dataset and in the validation cohort [*K* value: 0.60 (0.34–0.84)], which were approximately equal to the agreement between R1 and reference standard [*K* value: testing dataset: 0.48 (0.21–0.74); validation cohort: 0.63 (0.43–0.84)]but higher than the agreement between R2 and reference standard [*K* value: testing dataset: 0.30 (0.11–0.40); validation cohort: 0.45 (0.20–0.69)]. In addition, compared with the moderate agreement between R1 and R2 [*K* value: testing dataset: 0.58 (0.33–0.84); validation cohort: 0.55 (0.32–0.78)], the agreement was improved to substantial between R1 and automated segmentation-based assessment [*K* value: testing dataset: 0.85 (0.70–1.00); validation cohort: 0.74 (0.53–0.96)].

**Table 5:** The agreement of treatment response assessment

	Testing dataset	Validation cohort
R1 *vs.* reference standard	0.48 (0.21–0.74)	0.63 (0.43–0.84)
R2 *vs.* reference standard	0.30 (0.11–0.40)	0.45 (0.20–0.69)
Automated segmentation *vs.* reference standard	0.51 (0.23–0.79)	0.60 (0.34–0.84)
R1 *vs.* R2	0.58 (0.33–0.84)	0.55 (0.32–0.78)
R1 *vs.* Automated segmentation	0.85 (0.70–1.00)	0.74 (0.53–0.96)
R2 *vs.* Automated segmentation	0.46 (0.20–0.72)	0.50 (0.24–0.75)

R1: an attending radiologist with 8 year’s reading experience; R2: a fellow radiologist with 4 year’s reading experience

## Discussion

In this study, our results showed that the deep learning-based 3D U-Net can be trained to segment liver and liver metastases on DWI images and could subsequently reflect treatment response accurately according to the RECIST 1.1 in patients with liver metastases. The accuracy of the automated segmentation-based assessment was 0.74 in the validation cohort, and the AUC achieved 0.83. The output was comparable to an attending radiologist’s measurement but superior to a fellow radiologist.

The effects of therapies on patients with liver metastases are commonly evaluated with long and frequent imaging follow-ups. Measurement of size is a key element of MR interpretation as well as therapeutic decision-making. Reproducible measurements of size and optimization of them are therefore important. Several recent studies have shown that the size-based RECIST 1.1 criteria provide an accurate measure of response to targeted cancer therapy and which has been widely used in most clinical trials [16]. However, there has been some concern that RECIST may significantly underestimate or overestimate the disease progress due to poor agreement between observers on tumor quantity [17]. Therefore, an objective and accurate quantitative measurement of the lesions on both baseline and post-treatment examinations has practical value for full playing to improve the performance of RECIST.

An algorithm based on deep learning was proposed in this study for segmenting the metastatic lesion and calculating the size of the tumor to overcome the limitations of manual tumor response assessment. Through reliable measurements of hepatic metastases, deep learning-based quantification might improve RECIST criteria performance. The application of deep learning-based algorithms for accurate and efficient organ and tumor segmentation has been widely reported, for example, myocardium segmentation [18], ventricle segmentation [19] and brain metastases segmentation [20, 21]. Many specified algorithms have been developed for liver and liver lesions segmentation [14, 22, 23]. Given that the main purpose of this study is to explore the application value of the deep learning model in the actual clinical practice, instead of exploring a new segmentation model. Therefore, in this study, we selected the traditional and classic 3D U-Net [24] model for liver and liver metastases segmentation, for which with stable segmentation performance. The 3D U-Net algorithm has previously been proven to achieve excellent segmentations of liver metastases [25], which is similar to our results.

In this study, we obtained satisfied liver metastases segmentation with a high DSC of 0.87 ± 0.07 in the testing dataset and 0.85 ± 0.08 in the validation dataset, which seems higher than the semi-automatic liver metastases segmentation on CT images performed by Eugene Vorontsov (DSC values of 0.14, 0.53, and 0.68 for the metastatic lesion smaller than 10 mm, 10–20 mm, and larger than 20 mm) [12].

Two reasons may be attributed to this. First, to segment liver metastases automatically, we developed a two-step deep learning-based 3D U-Net. The combination of the two 3D U-Nets could lead to efficient liver metastases by excluding the interference factors outside the liver, such as the bowel. Second, we chose DWI images as the input images for the segmentation model development. The signal intensity of metastatic lesions is very high compared with the surrounding liver parenchyma, and the lesion borders can be defined with exceptional precision when the vessel signal is suppressed.

In addition, high intraclass correlation coefficients among different radiologists for metastasis size measurement on DWI images have been reported compared with other sequences. Lestra et al. [9] compared different MRI sequences on the dimension measurement variability of liver metastases and concluded that DWI might be the most reliable MR sequence for monitoring size variations. Sankowski et al. [26] found that there was no significant difference between enhanced T1WI and DWI for the detection of liver metastases. Lavelle et al. [27] found that the reference standard and DWI showed an excellent agreement according to the RECIST evaluation. This is also the reason why the DWI sequence was selected in this study.

The precise automated segmentation of liver metastases lays a strong foundation for the subsequent RECIST 1.1 assessment. In the validation cohort, based on the automated segmentation of liver metastases, 24/31 pairs of examinations were correctly classified according to the RECIST criteria with an accuracy of 0.74 and AUC of 0.83, the consistency to manual segmentation-based assessment was moderate [K value: 0.60 (0.34–0.84)]. The results were superior to that of a fellow radiologist and comparable to that of a junior attending radiologist when measuring the same pairs of 31 scans. Among the 7 pairs of examinations mistakenly classified, 3 PD cases were defined as SD, 2 SD cases as PR, and 2 PR cases as PD. Reasons for these mistakes included a poor performance in tumor segmentation, errors in the selection of measurable targets, and intercurrent diseases.

Moreover, in this study, the accuracy and consistency of the response assessment in the testing dataset are lower than those of the validation cohort for both the radiologists and the deep learning-based model. The reason may be that the ratio of patients with more than 5 target lesions in the testing dataset was significantly higher than that in the validation cohort as shown in Table 2. This may indicate that the number of target lesions will affect the accuracy of treatment response assessment. However, restricted by the limited retrospective data, further subgroup analysis of the effect of number on assessment was not conducted here.

There were several limitations of our study. Firstly, our study has a limited sample size. Although the deep learning-based model provided satisfactory results for assessing tumor response in the testing and validation cohort, data from multiple centers and different centers are urgently needed to assess the robustness and reproducibility. Secondly, limited by the data size, subgroup analyses divided by the location of primary cancer, the number of target lesions, and the scanning vendors were not performed. Lastly, the whole data set was based on only one set of radiologists’ manual segmentations. Several independent manual segmentations of liver and liver metastases by different radiologists would be required to study the variability between and within observers.

In conclusion, using the deep learning-based liver metastases segmentation and the rule-based program could evaluate therapy response according to RECIST 1.1 criteria, with comparable results to the junior radiologist and superior to the fellow radiologist.

## Data Availability

The datasets used and/or analyzed during the current study are available from the corresponding author on reasonable request.

## Abbreviations

ADC: Apparent diffusion coefficient
AUC: Area under the curve
CR: Complete response
CT: Computed tomography
DCE: Dynamic contrast-enhanced
DSC: Dice similarity coefficient
DWI: Diffusion weighted imaging
HD: Hausdorff distance
MRI: Magnetic resonance imaging
PD: Progressive disease
PR: Partial response
RECIST: Response Evaluation Criteria in Solid Tumors
ROC: Receiver operating characteristics
SD: Stable disease
SD: Standard deviation
T1WI: T1-weighted imaging
T2WI: T2-weighted imaging
VS: Volumetric similarity
